# Antibody-Capped Mesoporous Nanoscopic Materials: Design of a Probe for the Selective Chromo-Fluorogenic Detection of Finasteride

**DOI:** 10.1002/open.201100008

**Published:** 2012-10-24

**Authors:** Estela Climent, Ramón Martínez-Máñez, Ángel Maquieira, Félix Sancenón, M Dolores Marcos, Eva M Brun, Juan Soto, Pedro Amorós

**Affiliations:** a Centro de Reconocimienro Molecular y Desarrollo Tecnológico (IDM), Unidad mixta Universidad Politécnica de Valencia Universidad de Valencia, Universidad Politécnica de Valencia, Departamento de QuímicaCamino de Vera s/n, 46022 Valencia (Spain); b CIBER de Bioingeniería, Biomateriales y Nanomedicina (CIBER-BBN), Campus Rio Ebro50018 Zaragoza (Spain); c Institut de Ciència dels Materials (ICMUV), Universidad de ValenciaP.O. Box 2085, 46071 Valencia (Spain)

**Keywords:** antibodies, finasteride, hybrid materials, MCM-41, molecular gates

## Abstract

The synthesis of capped mesoporous silica nanoparticles (MSN) conjugated with an antibody (AB) as a gatekeeper has been carried out in order to obtain a delivery system able to release an entrapped cargo (dye) in the presence of a target molecule (antigen) to which the conjugated antibody binds selectively. In particular, MSN loaded with rhodamine B and functionalized on the external surface with a suitable derivative of *N*-(*t*-butyl)-3-oxo-(5α,17β)-4-aza-androst-1-ene-17-carboxamide (finasteride) have been prepared (**S1**). The addition of polyclonal antibodies against finasteride induced capping of the pores due to the interaction with the anchored hapten-like finasteride derivative to give a MSN–hapten–AB nanoparticle **S1-AB**. It was found that the addition of capped material **S1-AB** to water solutions containing finasteride resulted in displacement of the antibody, pore uncapping and entrapped-dye release. The response of the gated material is highly selective, and only finasteride, among other steroids, was able to induce a significant uncapping process. Compared with finasteride, the finasteride metabolite was able to release 17 % of the dye, whereas the exogen steroids testosterone, metenolone and 16-β-hydroxystanozolol only induced very little release of rhodamine B (lower than 10 %) from aqueous suspensions containing sensing solid **S1-AB**. A detection limit as low as 20 ppb was found for the fluorimetric detection of finasteride. In order to evaluate a possible application of the material for label-free detection of finasteride, the capped material was isolated and stored to give final sensing solid **S1-AB-i**. It was found to display a similar behavior towards finasteride as to that shown by freshly prepared **S1-AB**; even after a period of two months, no significant loss of selectivity or sensitivity was noted. Moreover, to study the application for the detection of finasteride in biological samples, this “aged” material, **S1-AB-i**, was tested using commercially available blank urine as matrix. Samples containing 70 and 90 % blank urine were spiked with a defined amount of finasteride, and the concentration was determined using capped **S1-AB-i**. Recovery ranges from 94 % to 118 % were reached.

## Introduction

The design of delivery systems able to selectively release entrapped guests in the presence of target molecules is a new research field that has recently attracted great attention.^1^ Traditional delivery systems are based on organic polymers that usually release their cargo through diffusion-controlled processes or degradation of the polymeric matrix.^2^ As an alternative, in recent years, silica mesoporous supports have been used as inorganic scaffolds for the storage and controlled release of drugs and organic molecules. The unique properties of mesoporous silica materials, such as the presence of ordered tailor-made mesopores with stable structures, large surface areas, bio-compatibility and the possibility to include gate-like scaffoldings on the external surface for the design of nanodevices for on-command delivery applications, make these solids suitable supports for the development of carriers for cargo delivery.^3^ In these systems, mass transport can be controlled using molecular and supramolecular interactions or suitable physical stimuli.^4^ In particular, delivery of the cargo in gated materials have been reported using changes in pH,^5^ temperature,^6^ redox potential,3d, 7 light,^8^ and the presence of small molecules.^9^ However, despite these interesting examples, some of the described systems show disadvantages for their potential use in advanced applications, such as a lack of function in aqueous environments and the use of complex stimuli for mass transport control. Moreover, examples of controlled guest release in response to small molecules or biomolecules are still very rare. One of the most frequently used types of biomolecules for the development of gated hybrid materials are enzymes. The wide collection of available enzymes that can selectively catalyze a large number of different chemical reactions makes these systems very appealing for the design of sensitive and specific mesoporous silica nanoparticles (MSN)-based nanodevices.^10^ Also, in this field we and others have reported the use of oligonucleotides for the design of gated MSN for delivery applications.^11^

Furthermore, it is apparent from the literature that most of the reported gated materials have been designed toward the development of advanced drug-delivery systems, but very few examples of pore-blockage or pore-opening protocols for sensing applications have been reported.^9^ However, the design of such systems able to respond to the presence of target molecules is an attractive approach for the development of new sensing paradigms. The protocol involves the use of selective molecular recognition events that control the gate-like scaffolding. The addition of the solid to a solution containing the target molecule induces pore opening and delivery of a suitable dye. If the opening and dye release is a consequence of a selective interaction, the recognition event is translated into a selective optical response. This approach separates the recognition protocols from the signaling event making sensing independent of the stoichiometry of the host–guest complex and, in some cases, simultaneously displaying features of signal amplification.

Given our interest in the development of advanced bio-inspired strategies on inorganic materials for sensing applications using biomolecules, we recently combined the use of antigen–antibody interactions with the preparation of capped MSN able to selectively deliver an entrapped dye in the presence of sulfathiazole.^12^ In particular, we believe that the use of antibodies could be a promising approach for the design of custom-made nanodevices for controlled delivery specifically triggered by target guests. The possibility of selecting antibodies for a countless number of antigens, including low molecular weight targets, makes this approach highly appealing for a wide range of applications. Additionally, this approach opens the possibility of developing novel label-free immunoassay paradigms in which the properties of the reporter (cargo) could be selected at will. Inspired by these previous results, we report herein the preparation of antibody-capped MSN for the selective fluorogenic signaling of finasteride, a substance used in sport doping.

## Results and Discussion

### Design and synthesis of gated MSN

The incorporation of gate-like ensembles on mesoporous scaffolds has proven to be a suitable approach for the development of nanoscopic solids for mass-transport control and for studying the factors that could influence the design of gating functions. Additionally, as stated above, very few examples have been reported that use capped materials for the design of new sensing concepts. Moreover, in this particular field, the use of antibodies or other biomolecules as “biological keys” opens a wide range of possible opportunities for the design of new sensing protocols. In this study, we selected MSN from the MCM-41 family as the support. This is a suitable inorganic matrix that displays several appropriate characteristics, such as homogeneous porosity, high inertness and ease of functionalisation. Moreover, MCM-41 materials usually contain mesopores in the 2–3 nm range, which allows a rapid uptake and release of selected guests.

We focused our attention on the potential detection of finasteride, a 5α-reductase inhibitor. Finasteride is known to convert testosterone to the more potent androgen 5α-dihydrotestosterone, a class of drug that is used therapeutically to prevent prostate cancer, treat benign prostatic hyperplasia and male baldness. It has been reported that administration of inhibitors of 5α-reductase, such as finasteride, can complicate the evaluation of steroid profiles in sport doping control, causing erroneous results. The reason lies in the suppression of production and renal excretion of 5α-reduced metabolites of anabolic steroids that can produce false-negative doping-control results. In a previous work, an enzyme-linked immunosorbent assay for the detection of finasteride was reported.^13^ In this work, a collection of immunoreagents and antiserum were obtained from which we selected the best hapten–antiserum pair for the development of an antibody-capped mesoporous hybrid nanomaterial for the fluorimetric detection of finasteride (see below).

**Scheme 1:**
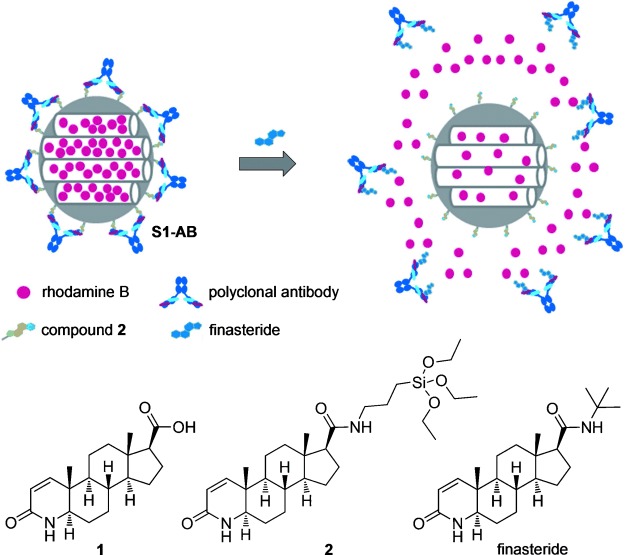
Representation of gated material S1-AB capped with an antibody, compounds 1 and 2 and the antigen, finasteride.

Scheme 1 shows the proposed paradigm using the gated support. Nanoparticles of mesoporous MCM-41 are first loaded with a fluorophore (rhodamine B), then, the external surfaces of the nanoparticles are functionalized with hapten-like derivative **2** to give solid **S1**. Finally the mesopores are capped with finasteride antibodies (**AB**) via interactions of the two binding IgG regions of the antibody with anchored **2**, giving solid **S1-AB**. Addition of the correspondent antigen, finasteride, induces uncapping of the pores and release of the entrapped fluorophore. The uncapping process is easily monitored by measuring the emission intensity of rhodamine B at 580 nm (*λ*_ex_=555 nm) in the aqueous phase.

For the preparation of solid **S1**, the hapten-like molecule **1** was selected. Capped materials obtained from **1** (via anchorage of silyl derivative **2**) proved to give a suitable response in terms of time and sensitivity (see below). Compound **2** was readily obtained through reaction of **1** with (3-aminopropyl)triethoxysilane. To prepare the final hybrid material **S1**, we made use of a two-step synthetic procedure. First, the mesoporous starting scaffold was added to a solution containing a high concentration of rhodamine B in order to reach an efficient loading of the pores. Second, organosilane derivative **2** was added. During the grafting of **2**, a high concentration of rhodamine B was contained in the solution, which inhibits diffusion of the dye from the pores into the solution and simultaneously hampers the entrance of **2** into the pores. Moreover, it has been reported that the reaction of organosilane derivatives with siliceous surfaces is usually quicker than diffusion processes from the pores.9a–c These features lead to the reasonable assumption that rhodamine B is captured within the mesopores and derivative **2** is anchored on the external surface of the final nanoparticles. Purple solid **S1** was isolated by filtration, washed with acetonitrile and dried at 35 °C for 12 h. Solid **S2** containing rhodamine B within the pores, but lacking derivative **2** was also prepared as a control.

Capping optimization with the specific polyclonal antibodies for finasteride was carried out by stirring solid **S1** (1 mg) for one hour in different aqueous dilutions of serum I (0.5 mL, pH 7.4; dilutions of 2.5:100, 1.25:100, 0.625:100 or 0.3125:100). After the capping process, the aqueous phase was removed by centrifugation, and the solid was washed twice to remove residual dye from the uncapped pores. The final material was suspended in water (0.025 mg mL^−1^; pH 7.4), and the kinetic release of rhodamine B was measured by monitoring the fluorescence emission at 580 nm (*λ*_ex_=555 nm). The most effective capping was observed with a 1.25:100 dilution of serum I. Lower proportions of serum showed only partial capping of the pores, which was reflected in significant rhodamine B release.

### Characterization of the hybrid materials

**Figure 1:**
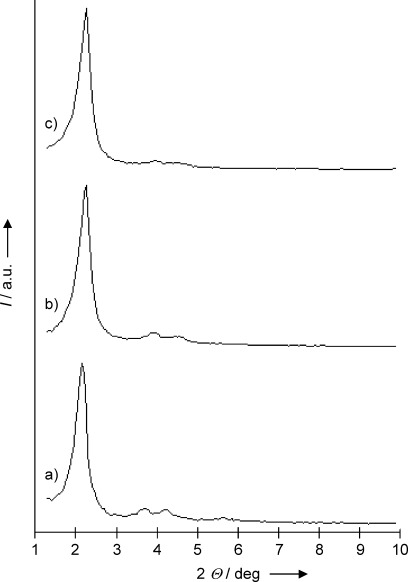
Powder X-ray patterns of the solids a) MCM-41, b) calcined MCM-41 and c) solid S1 containing rhodamine B and compound 2.

The prepared solids were characterized using standard techniques. Figure 1 shows powder X-ray diffraction (PXRD) patterns of synthesized MCM-41, calcined MCM-41, and **S1**. The PXRD of synthesized, siliceous nanoparticulated MCM-41 (Figure 1 a) shows the typical four low-angle reflections attributed to a hexagonal array that index as (100), (110), (200), and (210) Bragg peaks. From the PXRD data of synthesized MCM-41, a *d*_100_ spacing of 40.93 Å was calculated. A significant displacement of the (100) peak in the PXRD of the calcined nanoparticulated MCM-41 was found corresponding to an approximate cell contraction of 2 Å (Figure 1 b). This displacement and broadening of the (110) and (200) peaks are related to further condensation of silanol groups during the calcination step. Figure 1 c shows the PXRD patterns for solid **S1**. In this curve, the reflections (110) and (200) are less intense, most likely due to a reduction in contrast as a consequence of the pore loading with the dye and the functionalization with derivative **2**. Nevertheless, the clear presence of the (100) peak in the PXRD patterns indicates that the process of pore loading with rhodamine B and the additional functionalization with **2** did not modify the mesoporous structure of the MCM-41 support to a large extent.

**Figure 2:**
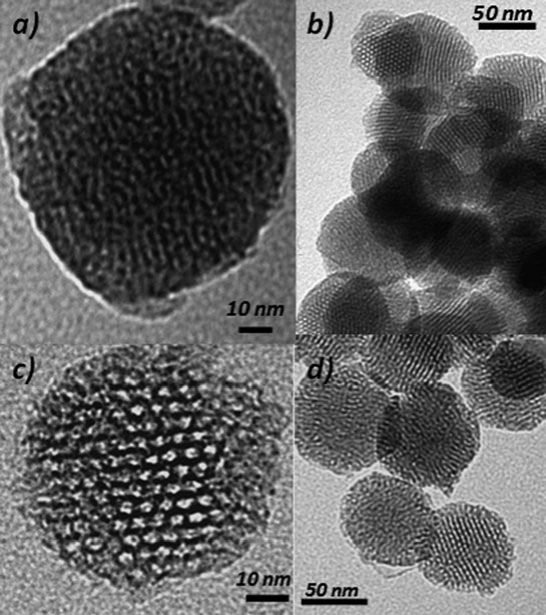
Transmission electron microscopy (TEM) images of a) solid S1-AB, b) solid S1 and c) and d) calcined MCM-41 sample showing the typical hexagonal porosity of the MCM-41 mesoporous matrix.

The mesoporous structure of the functionalized solid **S1** and in the capped material **S1-AB** of the mesoporous structure was also confirmed using transmission electron microscopy (TEM) analysis. The synthesized MCM-41 support was obtained as spherical particles with diameters ranging from 80 to 150 nm. Figure 2 shows TEM images for **S1** and **S1-AB**. In both cases, the typical hexagonal porosity and channels of the MCM-41 matrix as alternate black and white stripes are observed.

**Figure 3:**
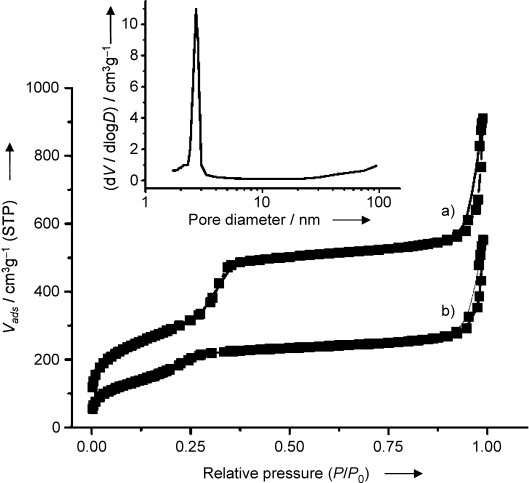
Adsorption–desorption isotherms for a) MCM-41 and b) dye-loaded and functionalized S1. Inset: Pore-size distribution of MCM-41.

The N_2_ adsorption–desorption isotherm of the calcined nanoparticulated MCM-41 is shown in Figure 3 a. A typical curve for mesoporous solids consisting of an adsorption step at intermediate *P*/*P*_0_ values (0.25–0.4) is observed. This curve corresponds to a type IV isotherm, in which the observed step indicates nitrogen condensation inside the mesopores. In the solids, the existence of uniform cylindrical mesopores—pore diameter of 2.62 nm and pore volume of 0.88 cm^3^ g^−1^, calculated using the Barret, Joyner and Halenda (BJH) model on the adsorption branch of the isotherm—is suggested by the absence of a hysteresis loop in this interval and the narrow BJH pore distribution (Table 1). The application of the Brunauer, Emmett and Teller (BET) model resulted in a value of 1053.4 m^2^ g^−1^ for the total specific surface. From the PXRD, porosimetry and TEM studies, the *a*_0_ cell parameter (4.57 nm), pore diameter (2.62 nm), and value for the wall thickness (1.95 nm) were calculated. In addition to this adsorption step associated to the micelle generated mesopores, a second feature appears in the isotherm at a high relative pressure (P/P_0_>0.85). This adsorption corresponds to the filling of the large voids among the particles (pore diameter of 36.4 nm and pore volume of 0.42 cm^3^ g^−1^, calculated by using the BJH model) and therefore must be considered as a textural-like porosity. The N_2_ adsorption–desorption isotherm of **S1** is shown in Figure 3 b. Solid **S1** presents a similar adsorption–desorption isotherm to the one observed with calcined MCM-41. However, the specific surface and pore volume of **S1** is reduced by ∼50 %, that is, a BJH mesopore volume of 0.44 cm^3^ g^−1^ and surface area of 625.2 m^2^ g^−1^ were calculated. This decrease in volume of adsorbed N_2_ and in pore size is ascribed to the presence of the dye and anchored derivative **2** on the external surface of **S1**. The moderate decrease on the specific surface area and pore volume in **S1** is most likely due to the fact that the pores are not completely closed. Additionally, the textural porosity is preserved strongly suggesting that dye molecules are placed inside the mesopores and not in the interparticle porosity.

**Table 1:** Brunauer, Emmett and Teller (BET)-specific surface values, pore volumes and pore sizes[a]

Solid	*S*_BET_ [m^2^ g^−1^]	Pore volume [cm^3^ g^−1^]	Pore size [nm]
MCM-41	1053.4	0.88	2.62
**S1**	625.2	0.44	2.33

[a] Calculated from N_2_ adsorption–desorption isotherms.

As described above, the transformation of **S1** to **S1-AB** by the addition of antibody does not significantly alter the structural order of the mesoporous support based on TEM images of this solid. Unfortunately, it was not possible to carry out additional PXRD or N_2_ adsorption–desorption studies on **S1-AB** due to the small amount of material prepared. However, despite this lack of complete characterization, the behavior of **S1-AB** is fully consistent with the capping–uncapping procedure described in Scheme 1 (see below).

The quantity of derivative **2** and dye in **S1** and dye in **S2** was determined by elemental analysis and thermogravimetric studies. The thermal analysis of **S1** and **S2** shows a typical behavior of functionalized mesoporous materials, that is, an initial weight loss between 25 and 150 °C related to solvent evaporation, a second loss between 150 and 800 °C due to the combustion of organic material, and a final loss in the 800–1000 °C range related to the condensation of the silanol groups. The dye content in solid **S1-AB** was determined from the difference between the amount measured for **S1** and the amount released into the aqueous washing fractions of the solid after interaction with the antibody (Table 2).

**Table 2:** Concentration [mmol g^−1^] of compound 2, rhodamine B and IgG in solids S1, S1-AB and S2

Solid	Compd 2	Rhodamine B	IgG
**S1**	0.275	0.389	–
**S1-AB**	0.275	0.298	0.0001926
**S2**	–	0.403	–

With the aim of estimating the amount of antibody in solid **S1-AB**, concentrations of immunoglobulin G (IgG, the most abundant type of antibody in plasma) were measured in serum I and in the aqueous solutions after the capping process by measuring the absorbance at 280 nm. Considering an average molecular weight (MW) of 150 kDa and the specific molar extinction coefficient of immunoglobulin, a concentration in serum I of 5.3 mg mL^−1^ was found. Taking into account the absorbance measurements, an estimated 87 % of the antibodies were incorporated in **S1-AB** when a serum dilution of 1.25:100 was used. With this data and the known concentration of IgG in serum I, it can be estimated that one gram of **S1-AB** contains 1.16×10^17^ immunoglobulin molecules. Additionally, considering the typical external surface of an MCM-41 support, an average distance between two immunoglobulin molecules of ∼17 nm is calculated (for details, see the Supporting Information). The amounts of compound **2**, antibody and rhodamine B in **S1**, **S1-AB** and **S2** are shown in Table 2.

### Functional antigen-driven controlled release

Several experiments were carried out in order to study the antigen-responsive controlled-release protocol using capped material **S1-AB** and finasteride as a trigger. The uncapping protocol, that is, the delivery of the entrapped dye from the pore voids to the aqueous solution, was observed with ease by monitoring the emission band of rhodamine B centered at 580 nm (*λ*_ex_=555 nm) in the aqueous phase. In a typical experiment, a suspension of **S1-AB** (0.2 mL, 2 mg mL^−^) was diluted with water (9.8 mL, pH 7.4) containing finasteride (130 ppm). A corresponding experiment was carried out under similar conditions using **S1-AB** but in the absence of finasteride. At a given time point, 1 mL of each suspension was filtered, and the emission of rhodamine B was measured at 580 nm.

**Figure 4:**
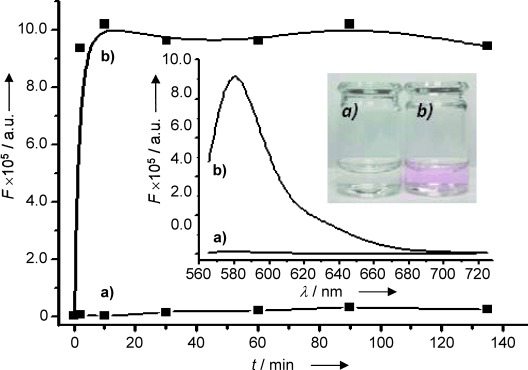
Fluorescence intensity over time of released rhodamine B at 580 nm (*λ*_ex_=555 nm) from S1-AB in water (pH 7.4) a) in the absence and b) in presence of of finasteride (130 ppm). The inset shows the fluorescence spectra of the rhodamine B-containing solution after filtration a) in absence and b) in presence of finasteride after 2 min. Fluorescence was measured after filtration (0.45 μm teflon filters). A photograph visualizes the colorimetric response.

From the kinetic-release curves shown in Figure 4, it can be seen that solid **S1-AB** was unable to release rhodamine B in the absence of finasteride, whereas in the presence of the antigen a remarkable delivery of the dye was found (cf. Figure 4 a and b). In this case, maximum delivery of dye was achieved after approximately 15 min. The release of rhodamine B was a direct consequence of the displacement process of the antibody from the outer surface of **S1-AB** induced by the presence of free finasteride. The observed uncapping process is caused by the stronger interaction of finasteride with the capping antibody compared with the interaction of the antibody and grafted **2**. In fact, when the same experiment is repeated in the presence of compound **1** instead of finasteride, only 23 % dye release is observed (compared with the amount of dye released in the presence of finasteride, i.e., 100 %).

Similar kinetic experiments with **S1-AB** using lower amounts of finasteride were carried out. In those cases, a lower amount of dye was released (rhodamine B delivery was proportional to the concentration, see below), and overall slower delivery kinetics were found. Finally, a release time of 75 min was selected for further experiments in order to reach a maximum dye delivery for all tested finasteride concentrations.

**Figure 5:**
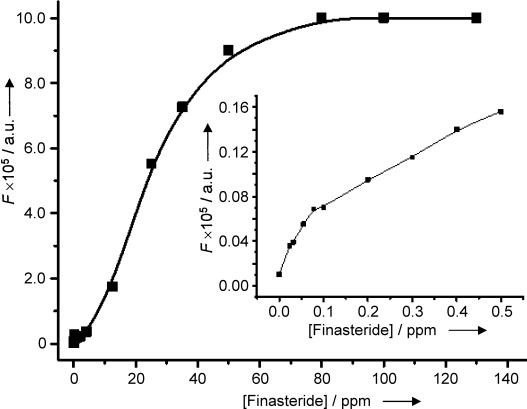
Fluorescence intensity of rhodamine B released from solid S1-AB measured at 580 nm (*λ*_ex_=555 nm) in water (pH 7.4) as a function of finasteride concentration after an incubation period of 75 min. Fluorescence was measured after filtration (0.45 μm teflon filters). The inset shows rhodamine B release in the low finasteride concentration range.

Figure 5 shows the fluorescence intensity of rhodamine B released from solid **S1-AB** measured in water (pH 7.4) as a function of the concentration of finasteride. The delivered amount of cargo is proportional to finasteride concentration displaying a typical noncompetitive immunoassay response curve, which is in accordance with an uncapping protocol based on the displacement of the antibody, as indicated above. From these studies, a detection limit for finasteride of 20 ppb was calculated.

### Selectivity studies

**Figure 6:**
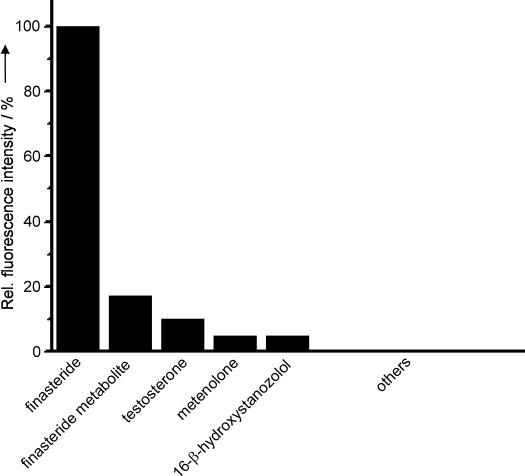
Relative fluorescence intensity of rhodamine B released from S1-AB was measured at 580 nm (*λ*_ex_=555 nm) in water (pH 7.4) in presence of exogen steroids finasteride, finasteride metabolite, testosterone, metenolone, 16-β-hydroxystanozolol, dutasteride, 1-testosterone, androstanolone (DHT), oxandrolone, or testosterone glucuronide (1 ppm). Fluorescence was measured after filtration (0.45 μm teflon filters).

It is well-known that antibodies can ideally identify and bind only to their unique antigen in complex mixtures. This property allows the design of highly selective capping systems that can ideally be opened using a unique “molecular key”. In order to investigate the selectivity in the opening protocol of our system, dye delivery from solid **S1-AB** was tested in the presence of other exogen steroids that produce similar effects to finasteride. The uncapping ability of these closely related molecules (at 1 ppm) is shown in Figure 6, which displays the fluorescence of the released dye from **S1-AB** after 75 min in the presence of the steroids finasteride, finasteride metabolite, testosterone, metenolone, 16-β-hydroxystanozolol, dutasteride, oxandrolone, 1-testosterone, androstanolone, or testosterone glucuronide (for chemical structures, see the Supporting Information). A very selective uncapping process in the presence of finasteride was observed. Only finasteride metabolite is able to release 17 % of the dye (relative to finasteride), whereas the exogen steroids testosterone, metenolone and 16-β-hydroxystanozolol induced a very small release of rhodamine B (lower than 10 %) from aqueous suspensions of the sensing solid **S1-AB**.

In addition to these delivery studies with **S1-AB**, further control experiments were carried out using solid **S2**, in order to evaluate the effect on the selective delivery process of the anchored hapten. Unlike **S1-AB**, solid **S2** contains rhodamine B in its mesopores but does not have any additional functionalization. Aqueous suspensions of solid **S2** (pH 7.5) alone showed a fast dye release. However in the presence of antibody, dye delivery was strongly inhibited due to unspecific adsorption of the antibody onto the surface of **S2**, most likely through interactions with the silanol groups on the silica mesoporous supports (SMPS). Very importantly, the addition of finasteride to **S2-AB** did not lead to cargo delivery, even when large amounts of finasteride were added (up to 200 ppm).

### Storage of sensing material and assays with urine standard

In order to improve the properties of **S1-AB** for a potential application in the detection of finasteride, we tried to isolate and store solid **S1-AB**. The final sensing material (**S1-AB-i**) was prepared from **S1-AB**, which was further dried in vacuo overnight and then stored at 4 °C. It was found that, even after a period of two months, stored solid **S1-AB-i** showed a similar sensing behavior toward finasteride as that shown by freshly prepared **S1-AB** without significant loss of selectivity or sensitivity.

Moreover, in order to study the possible application in the detection of finasteride in biological samples, **S1-AB-i** was tested using commercially available blank urine as matrix. In a first step, the tolerance of the solids to urine was studied. In a typical experiment, **S1-AB-i** (0.45 mg) was suspended in urine (5 mL), and the suspension was then diluted with varying amounts of water (pH 7.4). Following this procedure, calibration curves for finasteride containing 50, 70 and 90 % urine were obtained, and a similar linear range for all three tested dilutions was found. Moreover, it was confirmed that the results using solid **S1-AB-i** were similar to those obtained for **S1-AB**. In a second step, urine samples containing 70 and 90 % urine were spiked with finasteride, and the concentrations were determined using the previously obtained calibration curve. The results found are shown in Table 3. Target analyte spike recoveries in the range of 94–118 % were achieved.

**Table 3:** Finasteride concentrations [ppb] in samples containing 70 % and 90 % urine determined using solid S1-AB-i

Urine	Finasteride		Recovery
[%]	added	found	[%]
70	33.3	35.57±2.56	106.7
70	66.6	62.92±2.03	94.4
90	30	35.38±1.17	117.9
90	60	58.57±1.84	97.6

## Conclusion

In summary, the use of antibodies as gatekeepers on the surface of MSN provides a suitable tool for the design of delivery systems able to release entrapped guests in the presence of target molecules (antigen) to which an antibody binds selectively. In this work, we prepared MSN loaded with rhodamine B and functionalized with a suitable derivative of finasteride on their external surface. Addition of polyclonal antibodies for finasteride induced the capping of the pores due to the interaction with the anchored hapten-like derivative. This material was used for the fluorimetric recognition of finasteride and is one of the few examples that use a capped system and opening protocols for chemosensing applications. It was found that addition of the capped material to water solutions containing finasteride resulted in a displacement of the antibody, uncapping of the pores and release of the entrapped dye. The response of this gated material is highly selective, and only finasteride, among other steroids, was able to trigger dye delivery. A detection limit as low as 20 ppb was found for the fluorimetric detection of finasteride. Although this detection limit is higher than the value obtained using an enzyme-linked immunosorbent assay (ELISA, 0.01 ppb),^13^ we believe that gated SMPS based on the use of antibodies could be a promising route for the development of custom-made, controlled-delivery nanodevices specifically triggered by target molecular guests. The possibility of including antibodies as caps on diverse supports, the choice of the cargo molecule (e.g., various dyes and fluorophores), and the potential design of nanoparticles containing two or more different loaded signaling molecules capped with their corresponding antibodies open the door to the design of new multiplex label-free immunoassay protocols. Further studies in this field are ongoing. It is important to note that the use of capped nanoparticles introduces two remarkable advantages into the immunoassay developed format. One is the direct displayed response, which means that increasing the target concentration in samples results in an increase of the analytical signal. Secondly, for this format it is not necessary to synthesize the typical tracers used in ELISA or other immunoassay-labeled formats. In a wider context, this work suggests that the combination of SMPS with suitable binding sites is an excellent starting point for applying the versatility of supramolecular ideas to the design of nanoscopic devices, which is a promising approach for bringing molecular and supramolecular concepts to new advances in the field of nanoscience.

## Experimental Section

### General techniques

Powder X-ray diffraction (PXRD) and thermogravimetric analysis (TGA), elemental analysis, transmission electron microscopy (TEM), N_2_ adsorption–desorption, NMR, UV–visible (UV–vis) and fluorescence spectroscopy were employed to characterize the synthesized materials. PXRD measurements were performed on a D8 Advance diffractometer using Cu Kα radiation (Philips, Amsterdam, The Netherlands). Thermogravimetric analyses were carried out on a TGA/SDTA 851e balance (Mettler Toledo, Columbus, OH, USA), using an oxidizing atmosphere (air, 80 mL min^−1^) with a heating program: gradient of 393→1273 K at 10 °C min^−1^, followed by an isothermal heating step at 1273 °C for 30 min. TEM images were obtained with a 100 kV CM10 microscope (Philips). N_2_ adsorption–desorption isotherms were recorded with an ASAP2010 automated sorption analyzer (Micromeritics, Norcross, GA, USA). The samples were degassed at 120 °C in vacuo overnight. The specific surface areas were calculated from the adsorption data in the low pressure range using the Brunauer, Emmett and Teller (BET) model. Pore size was determined following the Barret, Joyner and Halenda (BJH) method. ^1^H and ^13^C NMR spectra were acquired with a Varian 300 spectrometer (Sunnyvale, CA, USA). UV–vis spectroscopy was carried out using a Lambda 35 spectrometer (PerkinElmer Instruments, Waltham, MA, USA). Fluorescence spectroscopy was carried out on a Felix 32 Analysis version 1.2 (Build 56, Photon Technology International, Birmingham, NJ, USA). The polyclonal-based antibodies for finasteride were obtained according to Ref. 13.

### Chemicals

Tetraethylorthosilicate (TEOS), *N*-cetyltrimethylammonium bromide (CTABr), NaOH, (3-aminopropyl)triethoxysilane, rhodamine B, finasteride, chemical reagents for hapten derivative synthesis (*N*,*N*′-dicyclohexylcarbodiimide and *N*-methylsuccinimide), bovine serum albumin (BSA), complete and incomplete Freund’s adjuvant and Tween 20 were purchased from Sigma–Aldrich Química (Madrid, Spain). Dutasteride was purchased from AK Scientific (Union City, CA, USA). (5α-17β)-3-Oxo-4-aza-androst-1-ene-17-carboxylic acid (**1**) was purchased from Steraloids Inc. (Newport, USA). Keyhole limpet hemocyanin (KLH) was purchased from Pierce Biotechnology (Rockford, IL, USA). Phosphate-buffered saline (PBS, pH 7.4, 20 mm) was prepared containing the following salts: Na_2_HPO_4_ (16.06 mmol L^−1^), KH_2_PO_4_ (2.92 mmol L^−1^), NaCl (274 mmol L^−1^) and KCl (5.46 mmol L^−1^). PBS (20 mm) was supplemented with Tween 20 (0.05 %) to give Tween 20-containing PBS (PBST). All products were used as received.

### Synthesis

***N*****-(3-triethoxysilyl(propyl))-3-oxo-(5α,17β)-4-aza-androst-1-ene-17-carboxamide (2)**: A modification of the active ester method for the amidation reaction between compound **1** and (3-aminopropyl)triethoxysilane was used.^14^ A mixture of *N*-hydroxysuccinimide (NHS, 90.6 mg, 0.79 mmol) and *N*,*N*′-dicyclohexylcarbodiimide (DCC, 162.4 mg, 0.79 mmol) in anhyd *N*,*N*-dimethylformamide (DMF, 1.0 mL) was added to a solution of **1** (238.06 mg, 0.75 mmol) in anhyd DMF (4 mL). The mixture was stirred at RT for 20 h, and precipitated dicyclohexylurea (DCU, white solid) was removed by centrifugation. (3-Aminopropyl)triethoxysilane (150 μL, 0.7 mmol) was added to the solution, and the reaction mixture was stirred at RT for 24 h. The solvent was evaporated in vacuo to give a yellow sticky oil. In order to eliminate traces of DCU, CH_2_Cl_2_ (1 mL) was added, and the solution was filtered through a short silica gel column. The final product was isolated as a colorless oil (0.302 g, 0.58 mmol, 83 %): ^1^H NMR (300 MHz, CDCl_3_): *δ*=0.61 (t, 2 H, C*H_2_*Si), 0.96 (s, 3 H, C*H_3_*C), 1.21 (t, 9 H, SiOCH_2_C*H_3_*), 1.24–2.11 (m, 22 H), 3.25 (t, 2 H, NHC*H_2_*CH_2_CH_2_Si), 3.80 (q, 6 H, CH_3_C*H_2_*OSi), 5.78 (d, 1 H, COC*H*=CH), 6.78 ppm (d, 1 H, COCH=C*H*); ^13^C NMR (75 MHz, CHCl_3_): *δ*=7.3 (*C*H_2_Si), 11.9 (*C*H_3_), 13.0 (*C*H_2_CH_2_Si) 13.3 (*C*H_3_), 18.2 (3C, *C*H_3_CH_2_O), 21.1, 24.9, 25.6, 25.9, 29.4, 30.6, 33.8, 35.3, 36.2, 36.6, 39.4 (NH*C*H_2_CH_2_CH_2_Si), 48.8, 55.5, 58.2 (3C, CH_3_*C*H_2_O), 59.5, 122.9 (CO*CH*=CH), 158.4 (COCH=*CH*), 164.9 (*C*ONHCH), 173.7 ppm (*C*ONHCH_2_).

**MCM-41 mesoporous nanoparticles**: NaOH (2.00 mol L^−1^, 3.5 mL) was added to a solution of CTABr (1.00 g, 2.74 mmol) in deionized H_2_O (450 mL). After the solution temperature was adjusted to 80 °C, TEOS (5.00 mL, 2.57×10^−2^ mol) was added dropwise to the surfactant solution. The mixture was stirred for 2 h to give a white precipitate. The solid was isolated by centrifugation and washed with deionized H_2_O and EtOH (95:5) and then dried at 60 °C for 12 h to give MCM-41. In order to remove the template phase, MCM-41 was calcined at 550 °C for 8 h using an oxidizing atmosphere (air).

**Solid S1**: In a typical synthesis, calcined MCM-41 (0.3 g) and rhodamine B (0.115 g, 0.24 mmol) were suspended in CH_3_CN (35 mL) and heated at 120 °C in a Dean–Stark apparatus to remove adsorbed H_2_O by azeotropic distillation under an inert Ar atmosphere. After removing 10 mL, the suspension was stirred at RT for 24 h for loading of the MCM-41 pores. An excess of compound **2** (0.2 g, 0.384 mmol) in CHCl_3_ (0.5 mL) was added, and the final mixture was stirred at RT for 5.5 h. Resulting solid **S1** was isolated by filtration, washed with CH_3_CN (10 mL) and dried at 38 °C for 12 h.

**Solid S1-AB**: In order to prepare solid **S1–AB** and study the interactions between the anchored hapten **2** and the surface of solid **S1**, and the specific polyclonal-based antibodies for finasteride, portions of solid **S1** (1 mg) were suspended in varying aq dilutions of serum I (0.5 mL, pH 7.4; dilutions: 2.5:100, 1.25:100, 0.625:100, and 0.3125:100). Each suspension was stirred at RT for 1 h. After the capping process, the aq phase was removed by centrifugation to eliminate residual dye and free antibody, and the concentration of immunoglobulin (IgG) in the removed aq phase was measured. Results are shown in Table 4. Taking into account the results shown in Table 2, the ideal aq dilution for the preparation of the final material **S1-AB** was 1.25:100 of serum I (pH 7.4). Lower concentrations lead to incomplete pore capping, whereas higher concentrations of serum I lead to complete pore capping and undesired antibody adsorption.

**Table 4:** Amounts of serum I used in the capping process to generate S1-AB, and the corresponding calculated amount of antibody IgG

		Serum I		IgG
Assay	added[a]	found[b]	retained[c] [%]	[μmol g^−1^]
1	0.025	0.0057	77.2	0.3418
2	0.0125	0.0016	86.9	0.1926
3	0.00625	–	100	0.1107
4	0.003125	–	100	0.0553

[a] Amount of serum I as a dilution factor in H_2_O added to a suspension of **S1**.

[b] Amount detected in the aq phase after incubation with **S1** for 1 h (capping process).

[c] Amount (%) of serum I retained on the surface of **S1**, giving **S1-AB**.

[d] Calculated concentration of immunoglobulin (IgG) in the removed aq phase after capping, based on a serum I IgG concentration of 5.3 mg mL^−^, an average molecular weight for IgG of 150 kDa and a molar extinction coefficient at 280 nm of 210 000 M^−1^ cm^−1^.

## Supplementary material

Detailed facts of importance to specialist readers are published as ”Supporting Information”. Such documents are peer-reviewed, but not copy-edited or typeset. They are made available as submitted by the authors.
