# Supplementary material for: Antibody-Capped Mesoporous Nanoscopic Materials: Design of a Probe for the Selective Chromo-Fluorogenic Detection of Finasteride
Source: ChemistryOpen. 2012 Oct 24;1(6):251–9. doi: 10.1002/open.201100008 (PMC3922482; doi:10.1002/open.201100008)
Supplement: Supplementary file 1 [file open0001-0251-SD1.pdf]

ChemistryOPEN

Supporting Information

© 2012 The Authors. Published by Wiley-VCH Verlag GmbH & Co. KGaA, Weinheim

Antibody-Capped Mesoporous Nanoscopic Materials: Design of a Probe for the Selective Chromo-Fluorogenic Detection of Finasteride

Estela Climent,[a, b] Ramón Martínez-Máñez,*[a, b] Ángel Maquieira,*[a] Félix Sancenón,[a, b] M. Dolores Marcos,[a, b] Eva M. Brun,[a] Juan Soto,[a] and Pedro Amorós[c]

open_201100008_sm_miscellaneous_information.pdf

Chemical structures of steroids tested during selective studies.

Chemical structures of finasteride, dutasteride, finasteride metabolite, metenolone, oxandrolone, 16-β-hydroxystanozolol, 1-testosterone, androstanolone, testosterone and testosterone glucuronide are showed in Figure SI-1.

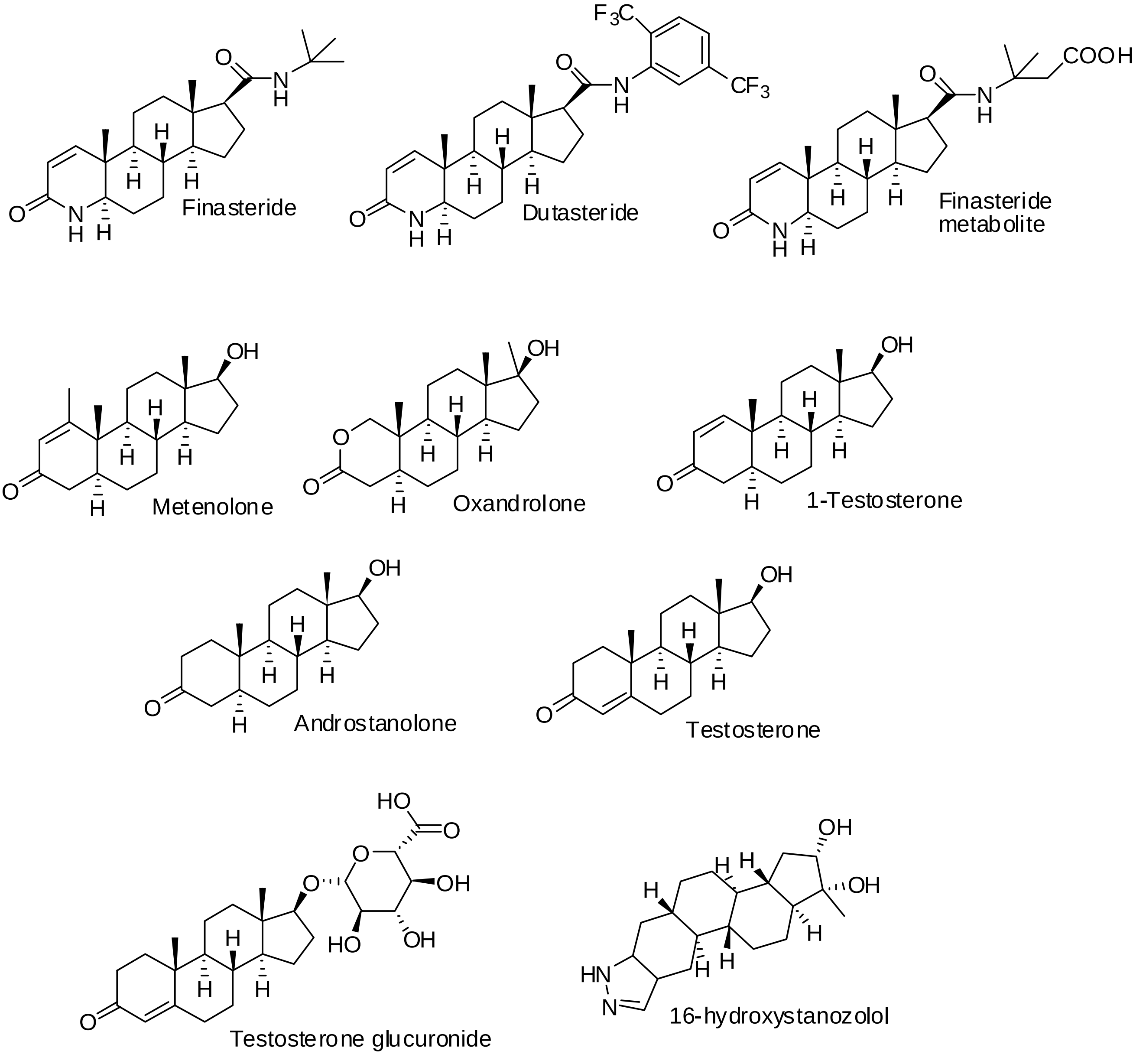

Figure SI-1. Chemical structures of substances tested during studies of selectivity.

Calculation of the average distance between immunoglobulin molecules.

With the aim of estimate the content of the antibody in the solid **S1-AB**, concentrations of IgG immunoglobulin (the most abundant type of antibody in plasma) were measured in serum I and in the aqueous solutions after the capping process via monitorization of the absorbance at 280 nm. Bearing in mind a typical MW for immunoglobulin of 150000 Da and the corresponding extinction molar coefficient of immunoglobulin, a content of 5.3 mg of IgG / mL of serum I was found. Taking into account the absorbance measurements, we can estimate than ca. 87% of the antibodies were incorporated in **S1-AB** when a dilution of sera of 1.25/100 was used. With this data, and knowing the concentration of IgG in serum I, it can be estimated that **S1-AB** material contains 1.16 x 10^{17} immunoglobulin molecules/g of solid using the following equation:

$$\frac{5.3\ \text{g IgG}}{1\ \text{L serum}} \bullet \frac{1.25\ \text{L serum}}{100\ \text{L solution}} \bullet \frac{0.5\ \text{L solution}}{1\ \text{g solid}} \bullet \frac{1\ \text{mol IgG}}{150000\ \text{g IgG}} \bullet \frac{6.022\ \text{x}\ 10^{23}\ \text{molecules IgG}}{1\ \text{mol IgG}} \bullet \frac{87\ \text{molecules IgG retained}}{100\ \text{molecules IgG}} = \frac{1.16\ \text{x}\ 10^{17}\ \text{molecules IgG retained}}{1\ \text{g solid}}$$

Additionally, and considering the typical external surface of an MCM-41 support (ca. 35 - 100 nm), an average distance between two immunoglobulin molecules of ca. 17 – 29 nm is calculated applying the following equation:

$$\frac{1}{\sqrt{\frac{1.16\ \text{x}\ 10^{17}\ \text{molecules IgG retained}}{1\ \text{g solid}} \bullet \frac{1\ \text{g solid}}{(35-50)\ \text{x}\ 10^{10}\ \text{nm}^2}}} = \frac{17-29\ \text{nm}}{1\ \text{molecule IgG}}$$
